# Sex Differences in Kappa Opioid Receptor Agonist Mediated Attenuation of Chemotherapy-Induced Neuropathic Pain in Mice

**DOI:** 10.3389/fphar.2022.813562

**Published:** 2022-02-18

**Authors:** Kelly F. Paton, Dan Luo, Anne C. La Flamme, Thomas E. Prisinzano, Bronwyn M. Kivell

**Affiliations:** ^1^ School of Biological Sciences, Centre for Biodiscovery, Victoria University of Wellington, Wellington, New Zealand; ^2^ Department of Pharmaceutical Sciences, University of Kentucky, Lexington, KY, United States; ^3^ Malaghan Institute of Medical Research, Wellington, New Zealand

**Keywords:** paclitaxel, kappa opioid receptor, salvinorin A, chemotherapy-induced neuropathic pain, sex differences

## Abstract

Chemotherapy-induced neuropathic pain is a common side effect for cancer patients which has limited effective treatment options. Kappa opioid receptor (KOR) agonists are a promising alternative to currently available opioid drugs due to their low abuse potential. In the current study, we have investigated the effects of Salvinorin A (SalA) analogues, 16-Ethynyl SalA, 16-Bromo SalA and ethyoxymethyl ether (EOM) SalB, and in a preclinical model of paclitaxel-induced neuropathic pain in male and female C57BL/6J mice. Using an acute dose-response procedure, we showed that compared to morphine, 16-Ethynyl SalA was more potent at reducing mechanical allodynia; and SalA, 16-Ethynyl SalA, and EOM SalB were more potent at reducing cold allodynia. In the mechanical allodynia testing, U50,488 was more potent in males and SalA was more potent in females. There were no sex differences in the acute cold allodynia testing. In the chronic administration model, treatment with U50,488 (10 mg/kg) reduced the mechanical and cold allodynia responses to healthy levels over 23 days of treatment. Overall, we have shown that KOR agonists are effective in a model of chemotherapy-induced neuropathic pain, indicating that KOR agonists could be further developed to treat this debilitating condition.

## Introduction

Chemotherapy-induced peripheral neuropathy (CIPN) is a common side effect of treating cancer (Sisignano et al., 2014; Addington and Freimer, 2016) with 68% of chemotherapy patients reporting CIPN within the first month of treatment (Seretny et al., 2014). CIPN is often characterized by spontaneous tingling or burning pain, hypersensitivity to mechanical and cold stimuli, and numbness (Forman, 1990; Dougherty et al., 2004). CIPN can be very debilitating, significantly impacting the quality of life and independence of cancer sufferers (Beijers et al., 2014; Mols et al., 2014). Often CIPN is identified as the reason for limiting either the dose or length of chemotherapy treatment and in severe CIPN cases, chemotherapy may be terminated (Holmes et al., 1991; Rowinsky et al., 1993); however, CIPN may persist for months following cessation of chemotherapy (van den Bent et al., 1997). Chemotherapy drugs that induce CIPN include vinca alkaloids, platinum derivatives and taxanes (Jaggi et al., 2011; Sisignano et al., 2014; Ewertz et al., 2015). Paclitaxel is a taxane chemotherapeutic widely used to treat solid tumors such as ovarian, breast, cervical, prostate, non-small cell lung, gastric, head and neck, Kaposi’s sarcoma, and pancreatic cancers (Khanna et al., 2015).

The pathogenesis of paclitaxel-induced neuropathic pain involves dying-back axonal damage. This causes distal sensory axons to degenerate in the peripheral nervous system, and causes sensitization of nociceptive afferents leading to neuropathic pain symptoms in the hands and feet in a “stocking and glove”-type distribution (Forman, 1990; Dougherty et al., 2004). The American Society of Clinical Oncology clinical practice guideline states there are no recommended therapeutics for the prevention of CIPN as there is not sufficient or consistent evidence from any randomized placebo-controlled trials (Hershman et al., 2014; Loprinzi et al., 2020). For the treatment of established CIPN, the serotonin-norepinephrine reuptake inhibitor duloxetine is the only agent which is moderately recommended (Hershman et al., 2014; Loprinzi et al., 2020). Mu opioid receptor (MOR) analgesics, including hydrocodone, morphine, oxycodone, methadone, and fentanyl patches or tramadol are considered a third-line therapy (Finnerup et al., 2015; Grace et al., 2016); however, MOR agonists are still commonly used to treat CIPN, with a recent study finding that 97% of CIPN patients used opioid therapy (Shah et al., 2018).

MOR agonists are associated with many side effects and can induce hyperalgesia (Grace et al., 2016), respiratory depression (Pattinson, 2008; Dahan et al., 2010), tolerance (Chu et al., 2006; Uniyal et al., 2020), and addiction (Compton and Volkow, 2006). In comparison, kappa opioid receptor (KOR) agonists do not have rewarding effects (Vonvoigtlander et al., 1983), and are not associated with respiratory depression (Freye et al., 1983) or gastrointestinal transit (Porreca et al., 1984), and have potential to treat pain (Beck et al., 2019; Paton et al., 2020a). The naturally occurring KOR agonist, Salvinorin A (SalA), has been used as a chemical scaffold to produce analogues with greater metabolic stability and potency. We investigated two analogues with alterations at the carbon-16 position, 16-Ethynyl SalA and 16-Bromo SalA, and one analogue at the carbon-2 position, ethoxymethyl ether Salvinorin B (EOM SalB). We have previously shown that 16-Ethynyl SalA and 16-Bromo SalA have antinociceptive effects in preclinical models of pain in mice, have a longer duration of action than SalA, and have improved side effect profiles (Paton et al., 2020b). Therefore, in the current study we have assessed the effect of 16-Ethynyl SalA, 16-Bromo SalA and EOM SalB in mice with paclitaxel-induced neuropathic pain. Furthermore, the majority of preclinical studies of the paclitaxel-induced neuropathic pain model have used male animals (Naji-Esfahani et al., 2016); however, in chronic pain studies, women typically have increased pain sensitivity and higher prevalence of clinical pain (Mogil, 2012; Bartley and Fillingim, 2013), and respond differently to pain medications (Pieretti et al., 2016). Therefore, we sought to understand the sex differences in the progression of paclitaxel-induced neuropathic pain and the differences in KOR treatment outcomes.

## Materials and Methods

### Animals

Female and male C57BL/6J mice (8 + weeks old) were used for all experiments. Animals were bred and housed at the Victoria University of Wellington (VUW) Animal Facility, Wellington, New Zealand. Animals were originally sourced from the Jackson Laboratories (Bar Harbour, ME, United States). All animals were group-housed (maximum 5 mice/cage) in a temperature (20–22°C) and humidity (55%) controlled environment. The animals were maintained on a 12-h light/dark cycle with lights on at 7 a.m. Access to food and water was provided *ad libitum* except during experimental sessions. For all paclitaxel-induced neuropathic pain experiments, soft paper/pulp-based Carefresh Natural bedding (Masterpet, Lower Hutt, NZ) was used in the home cage to avoid any mechanical stimulation to the paw. Each cage had shredded nesting material as environmental enrichment.

All experimental procedures were undertaken during the light cycle and in presence of white noise. Animals were handled for at least 2 days before testing to acclimatise to handling and prevent stress during experimental procedures. Animals were habituated to the experimental room for 30 min each day. All procedures were carried out with the approval of the VUW Animal Ethics Committee (approval numbers 21480 and 25751). All procedures were carried out in agreement with the New Zealand Animal Welfare Act, 1999.

### Drug Preparation

SalA was isolated and purified from *Salvia divinorum* leaves and assessed for purity (>98%) using high-performance liquid chromatography (HPLC) (Munro and Rizzacasa, 2003; Tidgewell et al., 2004). The SalA analogues were synthesized as previously described (Prevatt-Smith et al., 2011; Riley et al., 2014) and tested for purity (>95%) with HPLC. The prototypical KOR agonist U50,488 was purchased from Sigma-Aldrich (St. Louis, MO, United States) and morphine sulphate from Hospira NZ Ltd (Wellington, New Zealand). The compounds were dissolved in a vehicle containing DMSO, Tween-80 (Sigma-Aldrich), and 0.9% saline at a ratio of 2:1:7, respectively. The compounds were delivered at a volume of 10 μl/g of weight via intraperitoneal (i.p.) injection and delivered at 5 μl/g via subcutaneous (s.c.) injection in the dose-response experiments. The KOR antagonist *nor*-BNI (Sigma-Aldrich) was dissolved in 0.9% saline and injected s.c. 24 h before testing to selectively antagonize the KOR, as earlier pre-treatment intervals have been shown to also antagonize the MOR (Endoh et al., 1992; Kishioka et al., 2013).

### Induction of Paclitaxel-Induced Neuropathic Pain

Paclitaxel (Taxol, Tocris Bioscience #RDS109750, Bristol, United Kingdom) was made fresh daily by dissolving in absolute ethanol, cremophor EL (Sigma-Aldrich) and 0.9% saline at a ratio of 1:1:18, respectively. Experimental procedures were as previously described (Deng et al., 2015; Paton et al., 2017; Atigari et al., 2020). Mice were administered paclitaxel 4 mg/kg i.p. injections on four alternate days to give a cumulative dose of 16 mg/kg. Mechanical and cold allodynia were assessed every second day to measure the progression of paclitaxel-induced effects. Mice were placed in transparent plastic chambers upon a metal mesh stand. After a 20 min habituation to the apparatus, each hind paw was measured in duplicate for each type of stimulation, always beginning with mechanical testing. On days with behavioural measurements and a paclitaxel dose, measurements were always taken before the administration of paclitaxel.

### Von Frey Filament Procedure

Mechanical allodynia was measured using a 20-piece set of Semmes Weinstein von Frey filaments (#58011, Stoelting, IL, United States) as previously described (Paton et al., 2017; Atigari et al., 2020). Filaments numbered from 2 to 9 were used, with testing always beginning with filament number 5. The filament was applied at a right angle to the plantar surface of the hind paw with enough force to produce a slight bend. The filaments were held for 3 s or until a positive withdrawal response was observed. Mechanical allodynia was measured using a simplified up-down method until 5 filaments had been administered (Bonin et al., 2014). Mechanical allodynia for each animal was calculated by averaging the paw withdrawal thresholds from duplicate values for each hind paw.

### Acetone Test

Using a 1 ml syringe, a bubble of acetone was administered to the plantar surface of the hind paw with care not to cause any mechanical stimulation. The amount of time the animal reacted to the stimulus was recorded for 60 s following application. A positive reaction was defined as elevating, licking, biting or shaking of the paw. Two measurements were taken for each hind paw alternately, with 5 min between consecutive applications. Cold allodynia for each animal was calculated by averaging the duration of time spent responding to the acetone across the 4 applications.

### Acute Dose-Response in Paclitaxel-Treated Mice

On day 15, the cumulative dose-response effects were assessed in the paclitaxel-treated mice using a within-animals design (Paton et al., 2017; Atigari et al., 2020). The KOR agonists, morphine, or equivalent volumes of the vehicle were administered via s. c. injection every 30 min at increasing concentrations to create cumulative doses, with the mechanical and cold allodynia measured 30 min following each dose. The effects were measured in each hind paw once for each dose.

### Chronic Administration of Treatment in Mice With Paclitaxel-Induced Neuropathic Pain

The efficacy and tolerance effects of chronic administration of the KOR agonists were measured in mice with established paclitaxel-induced neuropathic pain. Following the measurements on day 15, animals were assigned to treatment groups to ensure an equivalent average mechanical allodynia score across all groups. The experimenter was blinded to the treatments each animal received. The doses used were based on the ED_80_ value obtained from the mechanical allodynia dose-response results. Animals were given daily i.p. injections starting on day 16. The treatments were as follows: 16-Ethynyl SalA, 3 mg/kg; 16-Bromo SalA, 4 mg/kg; U50,488, 10 mg/kg; morphine, 10 mg/kg; and vehicle. On all the even-numbered days the treatment was given 30 min before mechanical and cold allodynia testing.

### Statistical Analysis

GraphPad Prism (version 7.03, GraphPad Software, La Jolla, CA, United States) and SPSS Statistics (version 25, IBM, Armonk, NY, United States) were used to determine statistical significance. Values represented as the mean ± standard error of the mean (SEM) and were considered significant when *p* < 0.05. The data sets were tested for normality using the D’Agostino and Pearson omnibus normality test. Comparison of multiple treatment data was analyzed using one-way analysis of variance (ANOVA) followed by Bonferroni post-tests. Comparisons of multiple effects were analyzed using two-way ANOVA followed by Bonferroni post-tests. Two-way repeated-measures ANOVA was used when one variable was measured over time.

Dose-response data were analyzed by creating a non-linear regression. A four-parameter variable slope with least-squares ordinary fit was used to fit the curve to the data sets. For mechanical allodynia, the top constraint was set no more than 9.5. For cold allodynia, the bottom constraint was set at no less than 0. The extra sum-of-squares F test with the bottom, top, logED_50_ and hillslope parameters was used to compare the treatment curves, and with the null hypothesis that one curve fits all data sets. If the results showed a different curve fit for each data set, then the ED_50_ and E_max_ values were compared with one-way ANOVA analysis.

The effects of treatment, sex and time were analyzed with a three-way repeated-measures mixed ANOVA, with treatment and sex as between-subjects variables, and time as the within-subjects variable. The normality of the data was assessed with the Shapiro-Wilk test using the standardized residuals. The homogeneity of variances was measured using Levene’s test of equality of error variances. If the data was non-normal and had unequal variances at some time points, then the data was transformed. The sphericity of the data was tested using Mauchly’s test. If the *p* < 0.05, the assumption of sphericity was violated and the Greenhouse-Geisser correction was applied. The Bonferroni correction was applied for multiple families of comparisons and the adjusted α level reported.

## Results

**FIGURE 1:**
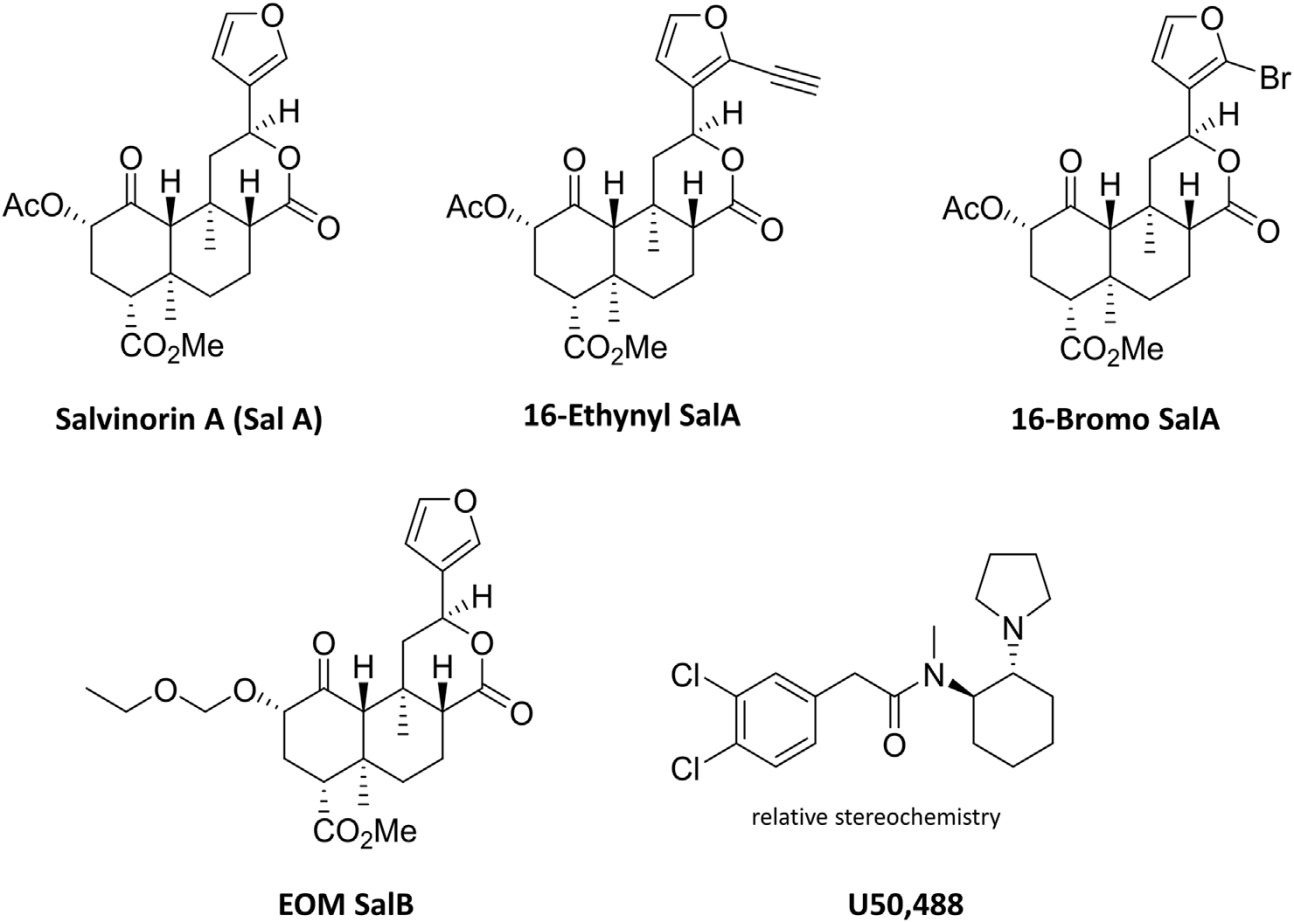
Chemical structures of Salvinorin A, 16-Ethynyl SalA, 16-Bromo SalA, EOM SalB, and U50,488.

**FIGURE 2:**
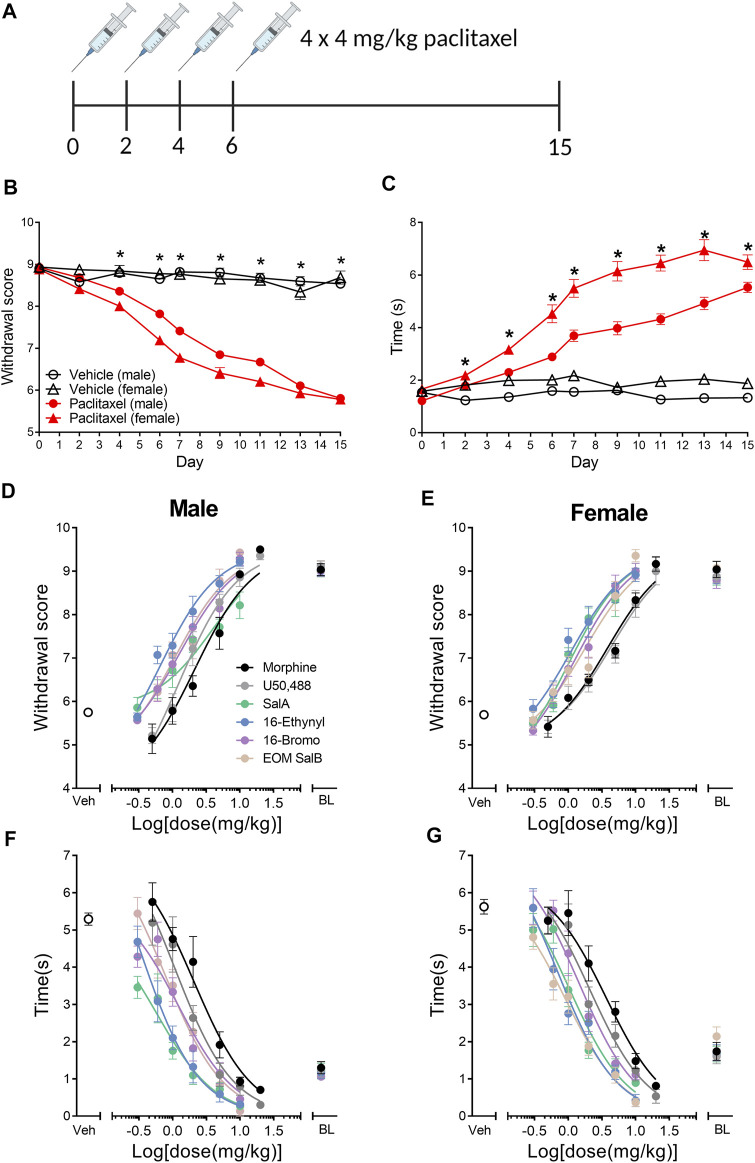
Paclitaxel administration produces mechanical and cold allodynia in male and female C57BL/6J mice. **(A)** Paclitaxel was administered as 4 doses of 4 mg/kg and allodynia measured until day 15. Numbers underneath the timeline represent experimental days. **(B)** Paclitaxel administration produced mechanical allodynia on days 4–15, shown as a reduction in withdrawal score measured using von Frey filaments. There were no sex differences in the withdrawal scores. **(C)** Paclitaxel administration had a significant effect on days 2–15, with an increase in reaction time to a cold acetone stimulus. There was an effect of treatment and sex, and but no interaction of both factors, with females showing an increased reaction time in the vehicle and paclitaxel treatment groups compared to males. Three-way repeated measures mixed ANOVA. *p < 0.006. Vehicle-treated *n* = 16–18; paclitaxel-treated males *n* = 50, females *n* = 36. **(D–G)** Dose-response effects of morphine and the KOR agonists comparing male and female mice with established paclitaxel-induced neuropathic pain. Mechanical allodynia was measured in **(D)** males and **(E)** females. Cold allodynia was measured in **(F)** males and **(G)** females. Veh refers to paclitaxel-treated animals treated with vehicle. BL refers to pre-paclitaxel baseline values. *n* = 6–8. Values presented as mean ± SEM. Image in panel **(A)** created using BioRender.com.

This study aimed to understand the effects of KOR agonists (Figure 1) for the treatment of paclitaxel-induced neuropathic pain in male and female mice. Initially, we investigated the sex differences throughout the progression of the paclitaxel-induced neuropathic pain model. A three-way repeated-measures mixed ANOVA was run to understand the effects of treatment, sex, and time on the mechanical withdrawal thresholds. The three-way interaction of treatment, sex and time was not statistically significant [F_(6.183,717.2)_ = 2.053, *p* = 0.055] (Figure 2A). There was a statistically significant two-way interaction between treatment and time [F_(6.183,717.2)_ = 66.771, *p* < 0.0005] and between treatment and sex [F_(1,116)_ = 9.744, *p* = 0.002]. Statistical significance of a simple main effect was accepted at a Bonferroni-adjusted alpha level of 0.006 due to multiple families of comparisons. There was a statistically significant simple main effect of treatment at days 4–15 (*p* < 0.006). The simple main effects of sex were not significant on any day. Overall, this shows that the paclitaxel treatment group was significantly different to the vehicle treatment group, but there were no sex differences.

A three-way repeated-measures mixed ANOVA was run to understand the effects of treatment, sex and time on the reaction times to the cold acetone stimulus. The three-way interaction was statistically significant [F_(6.618,767.7)_ = 3.284, *p* = 0.002] (Figure 2B). There was not a significant simple two-way interaction of treatment and sex at any time point (*p* > 0.006), however, there was a significant main effect of both treatment and sex on days 2–15 (*p* < 0.006). Overall, this means that there is an effect of paclitaxel treatment and an inherent difference between the sexes reaction to the cold stimulus, however, the effect of paclitaxel on each sex does not change over time.

### Cumulative Dose-Response Effects of Kappa Opioid Receptor Agonists

The dose-response effects of the KOR agonists were measured to understand and compare the potency and efficacy of each drug in both sexes. All of the curves were analyzed separated for sex, showing that a different curve fits each data set for the treatment of mechanical [F_(22,450)_ = 9.915, *p* < 0.0001] and cold allodynia [F_(22,450)_ = 13.33, *p* < 0.0001] (Figures 2D–G). The potencies (ED_50_ values) were compared by treatment and sex (Table 1). For mechanical allodynia, treatment with U50,488 in males was more potent than females (*p* = 0.0136), whilst the opposite was found with SalA, and with treatment in females significantly more potent than males (*p* = 0.0040). Morphine, 16-Ethynyl SalA, 16-Bromo SalA, and EOM SalB had no significant difference between the sexes. When the male treatment groups were compared to morphine, only 16-Ethynyl SalA was significantly more potent (*p* = 0.0152). When compared to the female morphine treatment group, SalA (*p* = 0.0098) and 16-Ethynyl SalA (*p* = 0.0242) were significantly more potent.

**TABLE 1:** Dose-response effects of the KOR agonists in female and male mice with established paclitaxel-induced mechanical allodynia. The potency (ED_50_) of the opioid receptor agonists were measured in mice of both sexes. U50,488 had more potent effects in males compared to females, whereas SalA was more potent in females. When the KOR treatments for each sex were compared to morphine, 16-Ethynyl SalA was significantly more potent in males and females. SalA was more potent than morphine in females only. Non-linear regression analysis. Two-way ANOVA with Bonferroni post-tests. *n* = 6–7. n.s. = not significant, *p < 0.05, **p < 0.01.

Opioid receptor agonist	ED_50_ value (mg/kg)		logED_50_ ± SEM		Two-way ANOVA comparisons					
	Male	Female	Male	Female	Male vs female		Male vs. Male treated with morphine		Female vs. Female treated with morphine	
Morphine	2.16	3.83	0.33 ± 0.11	0.58 ± 0.07	>0.9999	n.s.	—		—	
U50,488	1.23	4.38	0.09 ± 0.11	0.64 ± 0.09	0.0255	*	>0.9999	n.s.	>0.9999	n.s.
SalA	3.94	0.99	0.60 ± 0.12	−0.003 ± 0.101	0.0078	**	>0.9999	n.s.	0.0186	*
16-Ethynyl SalA	0.65	1.08	−0.19 ± 0.12	0.03 ± 0.13	>0.9999	n.s.	0.0283	*	0.0447	*
16-Bromo SalA	1.27	1.25	0.10 ± 0.01	0.10 ± 0.08	>0.9999	n.s.	>0.9999	n.s.	0.1636	n.s.
EOM SalB	1.07	1.76	0.031 ± 0.11	0.25 ± 0.11	>0.9999	n.s.	>0.9999	n.s.	>0.9999	n.s.

For the treatment of cold allodynia, the two-way ANOVA found no interaction of sex and treatment [F_(5,450)_ = 0.329, *p* = 0.8955] (Table 2). Therefore, only the data with the combined sexes could be compared, showing SalA (*p* = 0.0034) and 16-Ethynyl SalA (*p* < 0.0001) had significantly more potent antinociceptive effects than morphine.

**TABLE 2:** Dose-response effects of the KOR agonists in female and male mice with established paclitaxel-induced cold allodynia. Non-linear regression analysis was used to calculate the potency (ID_50_) of the opioid receptor agonists in male and female mice. Two-way ANOVA showed there was no significant interaction of treatment and sex. Using the combined sex data, SalA and 16-Ethynyl SalA had more potent antinociceptive effects than morphine. The efficacy of the treatments were not significantly different. One-way ANOVA with Bonferroni post-tests. *n* = 6–7. n.s. = not significant, **p < 0.01, ****p < 0.0001.

Opioid receptor agonist	ID_50_ value (mg/kg)		logID_50_ ± SEM		ID_50_ value (mg/kg)	logID_50_ ± SEM	*p* value for ID_50_ compared to morphine	
	Male	Female	Male	Female	Sexes combined	Sexes combined	Sexes combined	
Morphine	2.06	3.64	0.31 ± 0.12	0.56 ± 0.10	2.71	0.43 ± 0.08	—	
U50,488	1.06	2.08	0.03 ± 0.17	0.32 ± 0.12	1.46	0.17 ± 0.10	0.3349	n.s.
SalA	0.60	1.03	−0.22 ± 0.19	0.01 ± 0.12	0.82	−0.09 ± 0.13	0.0011	**
16-Ethynyl SalA	0.31	0.71	−0.51 ± 0.20	−0.15 ± 0.14	0.48	−0.32 ± 0.12	<0.0001	****
16-Bromo SalA	1.20	1.44	0.08 ± 0.13	0.16 ± 0.11	1.31	0.12 ± 0.09	0.1607	n.s.
EOM SalB	0.77	0.90	−0.11 ± 0.14	−0.04 ± 0.12	0.83	−0.08 ± 0.09	0.0021	**

### Antagonism of the Kappa Opioid Receptor

**FIGURE 3:**
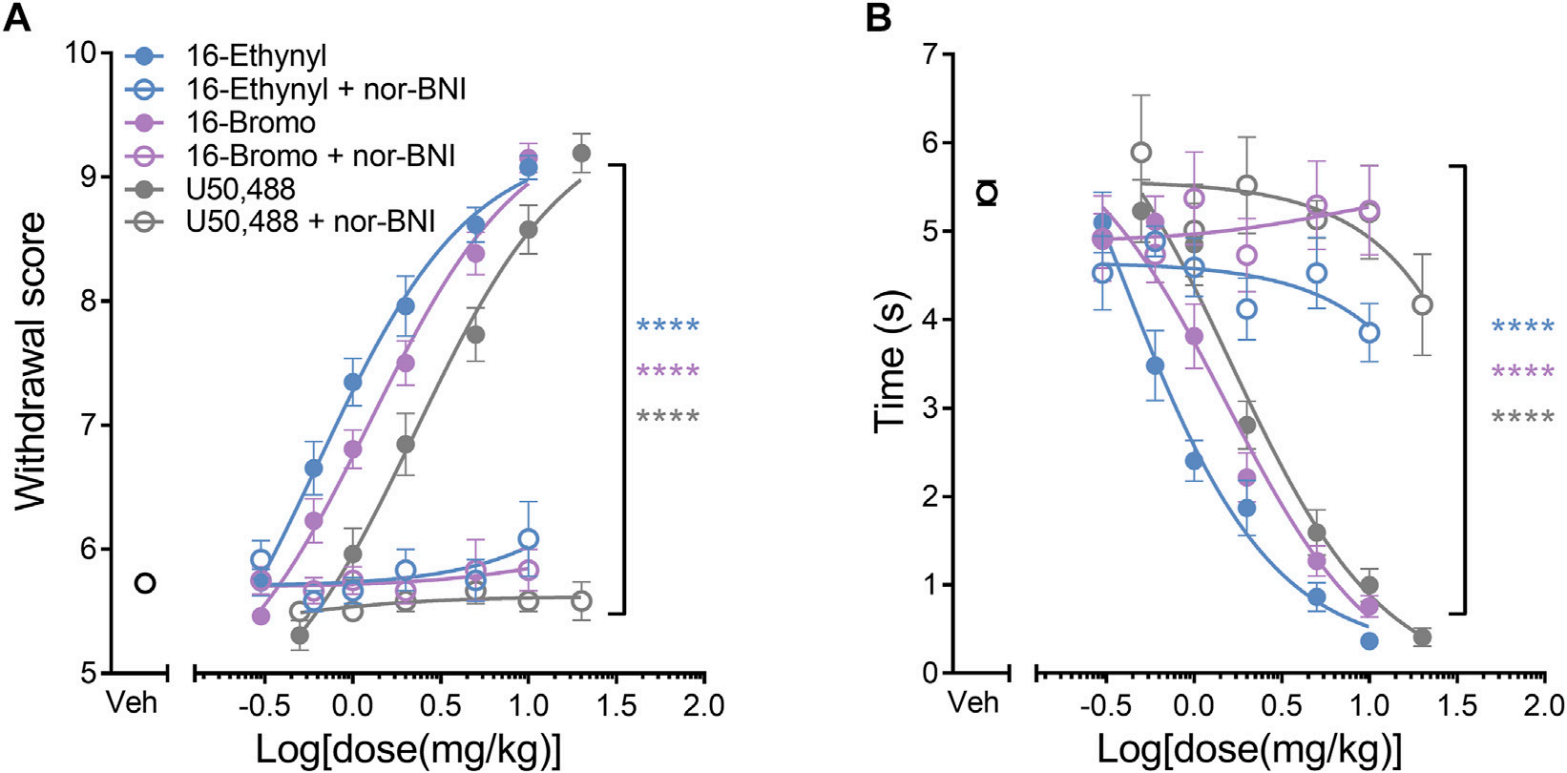
KOR antagonism reduces the antinociceptive effect of the KOR agonists. The selective KOR antagonist *nor-*binaltorphimine (*nor-*BNI, 10 mg/kg) was administered prior to the dose-response procedure. **(A)** Antinociceptive dose-response effects against mechanical allodynia. *Nor-*BNI reduced the antinociceptive effects of the KOR agonists at the highest dose for 16-Ethynyl SalA, 16-Bromo SalA (10 mg/kg) and U50,488 (20 mg/kg) to the mechanical stimulus. **(B)** Antinociceptive dose-response effects against cold allodynia. *Nor-*BNI reduced the antinociceptive effects of the KOR agonists to the cold stimulus. One-way ANOVA with Bonferroni post-tests. Values presented as mean ± SEM. *n* = 13 for KOR agonist treatment, n = 6 for groups with *nor-*BNI pre-treatment. ****p < 0.0001 indicates comparison between treatment with and without pre-treatment of *nor-*BNI.

16-Ethynyl SalA, 16-Bromo SalA, and U50,488 were antagonized at the KOR by pre-treating with *nor*-BNI (Figure 3). One-way ANOVA analysis of the values at the final dose for 16-Ethynyl SalA, 16-Bromo SalA (10 mg/kg), and U50,488 (20 mg/kg) showed a significant effect of treatment for the mechanical [F_(5,51)_ = 118.9, *p* < 0.0001] (Figure 3A) and cold allodynia data [F_(5,51)_ = 73.85, *p* < 0.0001] (Figure 3B). Bonferroni post-tests showed that there was a significant difference with pre-treatment of *nor*-BNI for all KOR agonists (*p* < 0.0001). The results show that the antinociceptive actions of the novel SalA analogues are mediated via the KOR.

### Effect of Chronic Administration of Kappa Opioid Receptor Agonists on Mechanical Allodynia

**FIGURE 4:**
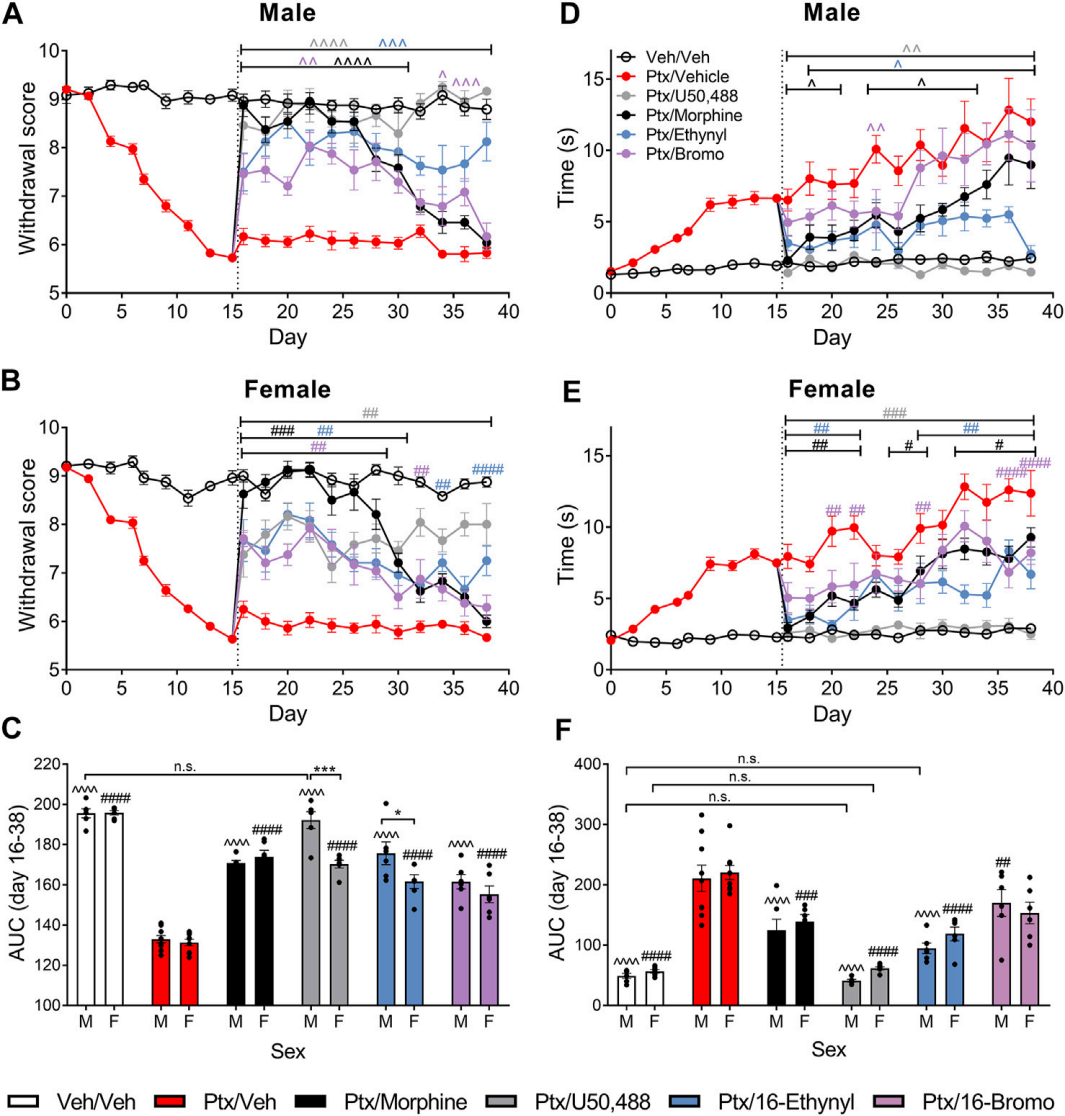
Chronic KOR treatment reduced mechanical and cold allodynia in mice with established paclitaxel (Ptx)-induced neuropathic pain. **(A, B)** Time course of the treatment effects of vehicle (Veh), morphine (10 mg/kg), U50,488 (10 mg/kg), 16-Ethynyl SalA (3 mg/kg), and 16-Bromo SalA (4 mg/kg) on mechanical allodynia in **(A)** males and **(B)** females. **(C)** Area under the curve (AUC) comparison within the male and female animals showed treatments all significantly increased the paclitaxel-induced withdrawal scores, with U50,488 treatment in males improving the mechanical thresholds to vehicle/vehicle levels. Comparison of the sex differences in the treatments found that U50,488 and 16-Ethynyl were more effective in males. **(D, E)** Time course of the treatment effects on cold allodynia in **(D)** males and **(E)** females. **(F)** AUC analysis showed that U50,488 treatment in both sexes and 16-Ethynyl treatment in males reduced the cold stimulus responding time to the same level as vehicle/vehicle controls. Two-way ANOVA with Bonferroni post-tests. n. s. = not significant, ^*p* < 0.05, ^  ^*p* < 0.01, ^  ^  ^*p* < 0.005, ^  ^  ^  ^*p* < 0,001 for male treatment group compared to male paclitaxel/vehicle group; ^#^ *p* < 0.05, ^##^ *p* < 0.01, ^###^ *p* < 0.005, and ^####^ *p* < 0.0001 for female treatment group compared to female paclitaxel/vehicle group; *p < 0.05, ***p < 0.005 for sex difference within treatment group. Values presented as mean ± SEM, *n* = 6−9.

We further assessed the effect of the KOR agonists using a chronic administration model, in which treatment began on day 16 post-initiation of paclitaxel-induced neuropathic pain. In male mice, 16-Ethynyl SalA and U50,488 reduced paclitaxel-induced mechanical allodynia on all days evaluated (*p* < 0.005; Figure 4A). Morphine treatment had antinociceptive effects on days 16–30, and 16-Bromo SalA on days 16–30 and 34–36 (*p* < 0.05; Figure 4A). In the female mice, U50,488 reduced mechanical allodynia at all time points evaluated, whereas, 16-Ethynyl SalA reduced mechanical allodynia at days 16–30, 34, and 38; 16-Bromo SalA at days 16–28, and 32; and morphine at days 16–30 (Figure 4C). The area under the curve (AUC) analysis showed that all treatment groups were significantly different to the paclitaxel/vehicle group within each sex (*p* < 0.001; Figure 4B). Furthermore, in the males, U50,488 treatment reduced the mechanical withdrawal thresholds to healthy control levels (vehicle/vehicle treatment group; *p* > 0.9999; Figure 4B). Further investigation into sex differences within each treatment showed that U50,488 and 16-Ethynyl SalA were more effective in male mice than female mice (*p* < 0.05), whereas, all other treatments had no sex differences (Figure 4C).

### Effect of Chronic Administration of Kappa Opioid Receptor Agonists on Cold Allodynia

In male mice, U50,488 reduced the paclitaxel-induced cold allodynia at all the days evaluated (days 16–38; *p* < 0.01); 16-Ethynyl SalA attenuated cold allodynia at days 18–38; 16-Bromo SalA at day 24; and morphine at days 16–20, 24–28, and day 32 (Figure 4D). In the female mice, U50,488 reduced the reaction time at all time points (days 16–38; *p* < 0.005); 16-Ethynyl SalA reduced cold allodynia at days 16–22 and 28–38 (*p* < 0.01); 16-Bromo SalA at days 20–22, 28 and 36–38 (*p* < 0.01); and morphine at days 16–22, 26–28, and 32–38 (*p* < 0.05; Figure 4E). In male mice, AUC analysis showed all KOR agonist treatments attenuated thermal nociception; in female mice, all treatment groups except 16-Bromo SalA attenuated thermal nociception when compared to the paclitaxel/vehicle control group (Figure 4F). U50,488 (both sexes) and 16-Ethynyl SalA (males only) returned antinociceptive responses to healthy control levels (vehicle/vehicle group). When the sexes were compared within each treatment, there were no significant sex differences (Figure 4F).

## Discussion

There is an urgent need to develop new treatments for CIPN, as this debilitating condition currently has very limited treatment options (Loprinzi et al., 2020). MOR agonists are often used to alleviate pain; however, these can potentiate pain when used chronically, and have addictive and aversive side effects (Chu et al., 2006; Compton and Volkow, 2006; Pattinson, 2008; Dahan et al., 2010; Roeckel et al., 2016). We have investigated the effect of KOR agonists for the treatment of paclitaxel-induced neuropathic pain due to the reduced abuse potential of KOR agonists, which is an important requirement for these treatments due to the long-term nature of chemotherapy regimens. We have further investigated the sex differences within our experiments due to the over-reliance on male animals used in research, which may not give an accurate representation of both sexes (Clayton and Collins, 2014; Lee, 2018; Shansky and Murphy, 2021).

We initially investigated the effect of sex on the onset of disease in the paclitaxel-induced neuropathic pain model. We showed that paclitaxel administration induces significant mechanical allodynia, with no sex differences at any time points. In contrast, measurements of cold allodynia showed the female mice had a longer reaction time in both the vehicle and paclitaxel groups. This is consistent with a previous study using NMRI mice, which found the paclitaxel-treated female mice had increased paw licking following cold stimulus to the paw between days 7–11, however, measurements on days 13 and 15 were not significantly different to males (Naji-Esfahani et al., 2016). The same study found no sex differences in the development of paclitaxel-induced mechanical allodynia measured using von Frey filaments (Naji-Esfahani et al., 2016). In further studies, there were no differences between the sexes in paclitaxel-induced mechanical allodynia in C57BL/6 mice (Smith et al., 2004); whereas in rats, there were both findings with no sex difference (Hwang et al., 2012) and with females showing greater mechanical hyperalgesia (Wang et al., 2018; Ferrari et al., 2020). Overall, the majority of studies have no inherent sex differences in paclitaxel-induced mechanical allodynia, whereas multiple studies have shown females to have a heightened cold response.

The antinociceptive dose-response effects of the KOR agonists were evaluated alongside morphine to assess the potency of the compounds in both sexes. The mechanical testing showed U50,488 was significantly more potent in males compared to females. It has been shown in previous studies that U50,488 exhibits higher antinociceptive potency in males when measured with the tail withdrawal assay (reviewed in Rasakham and Liu-Chen, 2011). However, in a similar paclitaxel-induced experiment performed in Sprague Dawley rats, acute morphine treatment (2–5 mg/kg i.p.) had the same antinociceptive effects in both sexes with mechanical allodynia (Hwang et al., 2012). We also found that SalA treatment was more potent in females. Interestingly, in rhesus macaques, SalA has sex differences in the pharmacokinetic effects, with females showing a slower elimination from plasma and a larger area under the concentration-time curve following intravenous injection (Schmidt et al., 2005), which may explain the increased antinociceptive effects produced in females. We also showed that 16-Ethynyl SalA was more potent than morphine for treatment of both mechanical and cold allodynia, which is similar to our previous study showing 16-Ethynyl SalA was more potent and efficacious than U50,488 in the warm water tail withdrawal assay (Paton et al., 2020b).

There are few previous studies measuring the effects of KOR agonists in a model of paclitaxel-induced neuropathic pain. We have shown the SalA analogue, β-tetrahydropyran SalB, and the mixed opioid receptor agonist MP1104 have anti-allodynic effects in this model (Paton et al., 2017; Atigari et al., 2020). In an alternative CIPN model, KOR agonist LOR17 was found to alleviate oxaliplatin-induced thermal hypersensitivity to a cold stimulus, and was more potent than U50,488 (Bedini et al., 2020). SalA has also been assessed in other models of neuropathic pain. SalA reduced pain in a sciatic nerve ligature model in male Wistar rats when injected directly into the insular cortex (Coffeen et al., 2018). Furthermore, an extract of *Salvia divinorum*, containing SalA, SalB, and other substances found in the leaves of the plant, reduced mechanical and thermal sciatic nerve ligature neuropathic pain when administered at 100–200 mg/kg i.p. (Simon-Arceo et al., 2017). The effect of KOR agonists in CIPN is an emerging area of research, however, these studies set the groundwork to show that KOR agonists have promise at treating neuropathic pain.

Several studies have assessed the effects of MOR agonists in the paclitaxel-induced neuropathic pain model. In male C57BL/6J mice, previous findings show morphine with an ED_50_ of 6.68 mg/kg against mechanical allodynia and 12.5 mg/kg against cold allodynia (Slivicki et al., 2018), whereas, we found that morphine was 3–6 fold more potent (mechanical allodynia ED_50_ of 2.16 mg/kg; cold allodynia ED_50_ of 2.06 mg/kg). The effects in both studies were done at 30 min post-injection, however, the dose-response in the Slivicki et al. (2018) study was done over multiple days rather than a cumulative dose-response in one session. Previous work has indicated that discrete versus cumulative dose-response procedures yield the same results (Schechter, 1997). A further difference between the studies is Slivicki et al. (2018) used an electronic von Frey anesthesiometer, whereas the current study used classical von Frey filaments of varying diameter. The electronic von Frey anesthesiometer may give more continuous data, as opposed to the individual von Frey filaments that each exert a discrete maximum force, and the electronic von Frey apparatus is believed to be more sensitive (Cunha et al., 2004).

In the current study, daily 10 mg/kg morphine administration was effective for 15 days against mechanical allodynia, whereas a previous study found morphine was only effective on the first treatment day and was no longer effective 3 days later (Slivicki et al., 2020). In male Sprague Dawley rats, Flatters and Bennett (2004) found an acute treatment of 4 mg/kg morphine was ineffective at treating paclitaxel-induced mechanical allodynia and 8 mg/kg only produced a 50% reversal of mechanical allodynia. A further study in male Sprague Dawley rats found that 4 mg/kg normalized the mechanical withdrawal thresholds to pre-paclitaxel baseline levels (Rahn et al., 2008). This shows there is great variation in the effects of morphine in the paclitaxel-induced neuropathic pain model. Reasons for variations in the results could include the use of different species (mice vs rats), different concentration of paclitaxel, the use of electronic vs. classical von Frey apparatus, and different experimental time points.

In the chronic administration regimen, we showed that U50,488 significantly reversed the effects of paclitaxel over 23 days, with no apparent tolerance effects. In the warm water (55°) tail withdrawal assay in C57BL/6 mice, U50,488 has been shown to cause tolerance effects, however, this was with an escalating dose scheme up to 75 mg/kg i.p. over 4 days (McLaughlin et al., 2004), whereas in the current study we used 10 mg/kg i.p. treatment daily. Interestingly, using a partial spinal nerve ligation model, phosphorylated KOR immunoreactivity was increased in the L4-5 dorsal horn regions of the spinal cord in male C57BL/6 mice and KOR knock-out mice there was increased mechanical allodynia and thermal heat hyperalgesia (Xu et al., 2004). However, due to this endogenous KOR activation in the mice with neuropathic pain, treatment with U50,488 showed increased tolerance compared to sham, and this tolerance effect was absent in prodynorphin or GRK3 knock-out mice (Xu et al., 2004). Furthermore, KOR antagonism with *nor-*BNI in mice and rats led to increased levels of mechanical and thermal allodynia (Obara et al., 2003). In comparing to the current study, because U50,488 does not show the tolerance effects associated with endogenous KOR activation, it could be that the endogenous KOR system is not activated to the same extent in the paclitaxel-induced neuropathic pain model compared to the partial spinal nerve ligation model; however, this effect has not been studied.

Interestingly, the KOR mediates the initial aversive component of paclitaxel-induced neuropathic pain (day 8), with an increase in prodynorphin levels in the nucleus accumbens (Meade et al., 2020). Due to this aversive nature of the pain, it is important to develop treatments that do not have negative side effects. We have previously shown that 16-Bromo SalA does not have anxiogenic effect in the elevated zero maze and the marble burying test; however, 16-Ethynyl SalA did significantly reduce exploratory behaviors in the elevated zero maze but had no effect in the marble burying test (Paton et al., 2020b). Furthermore, the sedative effects of the treatments should be considered, we know that 16-Ethynyl SalA, 16-Bromo SalA, and U50,488 have motor incoordination effects in the rotarod performance test (Paton et al., 2020b; Dunn et al., 2020); however, 16-Bromo SalA and 16-Ethynyl SalA did not reduce spontaneous locomotor activity at lower doses in rats (Riley et al., 2014). Even though the duration of action of these novel SalA analogues is longer than the parent compound (Paton et al., 2020b), the relatively short duration of action and negative side effects may hinder progression of these compounds into a clinical setting. However, these compounds show proof-of-concept that KOR agonists can be used for this form of neuropathic pain. In addition, there has been some progress in developing peripherally-restricted MOR agonists for the treatment of neuropathic pain (Tiwari et al., 2018), so further investigation into the mechanism of action could indicate whether a peripherally-restricted KOR agonist could be developed with no centrally-active side effects.

In conclusion, we have shown that KOR agonists have anti-allodynic effects in a mouse model of CIPN and are more potent than morphine for the treatment of paclitaxel-induced neuropathic pain. We have shown that U50,488 was more potent in male mice; whereas, SalA treatment was more potent in females. In the chronic administration paradigm, treatment with U50,488 reversed the paclitaxel-induced allodynia to healthy levels. Therefore, this study provides evidence that KOR agonists have potential for treating pain conditions associated with chronic neuropathy such as CIPN by reducing allodynia.

## Data Availability

The raw data supporting the conclusion of this article will be made available by the authors, without undue reservation.

## References

[B1] Addington J. Freimer M. (2016). Chemotherapy-induced Peripheral Neuropathy: an Update on the Current Understanding. F1000Res 5, F1000 Faculty Rev-1466. 10.12688/f1000research.8053.1 PMC4920214 27408692

[B2] Atigari D. V. Paton K. F. Uprety R. Váradi A. Alder A. F. Scouller B. (2021). The Mixed Kappa and delta Opioid Receptor Agonist, MP1104, Attenuates Chemotherapy-Induced Neuropathic Pain. Neuropharmacology 185, 108445. 10.1016/j.neuropharm.2020.108445 33383089 PMC8344368

[B3] Bartley E. J. Fillingim R. B. (2013). Sex Differences in Pain: a Brief Review of Clinical and Experimental Findings. Br. J. Anaesth. 111 (1), 52–58. 10.1093/bja/aet127 23794645 PMC3690315

[B4] Beck T. C. Hapstack M. A. Beck K. R. Dix T. A. (2019). Therapeutic Potential of Kappa Opioid Agonists. Pharmaceuticals (Basel) 12 (2), 95. 10.3390/ph12020095 PMC6631266 31226764

[B5] Bedini A. Di Cesare Mannelli L. Micheli L. Baiula M. Vaca G. De Marco R. (2020). Functional Selectivity and Antinociceptive Effects of a Novel KOPr Agonist. Front. Pharmacol. 11 (188), 188. 10.3389/fphar.2020.00188 32210803 PMC7066533

[B6] Beijers A. Mols F. Dercksen W. Driessen C. Vreugdenhil G. (2014). Chemotherapy-induced Peripheral Neuropathy and Impact on Quality of Life 6 Months after Treatment with Chemotherapy. J. Community Support. Oncol. 12 (11), 401–406. 10.12788/jcso.0086 25856013

[B7] Bonin R. P. Bories C. De Koninck Y. (2014). A simplified up-down method (SUDO) for measuring mechanical nociception in rodents using von Frey filaments. Mol. Pain 10 (1), 26. 10.1186/1744-8069-10-26 24739328 PMC4020614

[B8] Chu L. F. Clark D. J. Angst M. S. (2006). Opioid Tolerance and Hyperalgesia in Chronic Pain Patients after One Month of Oral Morphine Therapy: a Preliminary Prospective Study. J. Pain 7 (1), 43–48. 10.1016/j.jpain.2005.08.001 16414554

[B9] Clayton J. A. Collins F. S. (2014). Policy: NIH to Balance Sex in Cell and Animal Studies. Nature 509 (7500), 282–283. 10.1038/509282a 24834516 PMC5101948

[B10] Coffeen U. Canseco-Alba A. Simón-Arceo K. Almanza A. Mercado F. León-Olea M. (2018). Salvinorin A Reduces Neuropathic Nociception in the Insular Cortex of the Rat. Eur. J. Pain 22 (2), 311–318. 10.1002/ejp.1120 28975684

[B11] Compton W. M. Volkow N. D. (2006). Major Increases in Opioid Analgesic Abuse in the United States: Concerns and Strategies. Drug Alcohol Depend 81 (2), 103–107. 10.1016/j.drugalcdep.2005.05.009 16023304

[B12] Cunha T. M. Verri W. A. Jr. Vivancos G. G. Moreira I. F. Reis S. Parada C. A. (2004). An Electronic Pressure-Meter Nociception Paw Test for Mice. Braz. J. Med. Biol. Res. 37 (3), 401–407. 10.1590/s0100-879x2004000300018 15060710

[B13] Dahan A. Aarts L. Smith T. W. (2010). Incidence, Reversal, and Prevention of Opioid-Induced Respiratory Depression. Anesthesiology 112 (1), 226–238. 10.1097/ALN.0b013e3181c38c25 20010421

[B14] Deng L. Guindon J. Cornett B. L. Makriyannis A. Mackie K. Hohmann A. G. (2015). Chronic Cannabinoid Receptor 2 Activation Reverses Paclitaxel Neuropathy without Tolerance or Cannabinoid Receptor 1-dependent Withdrawal. Biol. Psychiatry 77 (5), 475–487. 10.1016/j.biopsych.2014.04.009 24853387 PMC4209205

[B15] Dougherty P. M. Cata J. P. Cordella J. V. Burton A. Weng H. R. (2004). Taxol-induced Sensory Disturbance Is Characterized by Preferential Impairment of Myelinated Fiber Function in Cancer Patients. Pain 109 (1-2), 132–142. 10.1016/j.pain.2004.01.021 15082135

[B16] Dunn A. Windisch K. Ben-Ezra A. Pikus P. Morochnik M. Erazo J. (2020). Modulation of Cocaine-Related Behaviors by Low Doses of the Potent KOR Agonist Nalfurafine in Male C57BL6 Mice. Psychopharmacology (Berl) 237 (8), 2405–2418. 10.1007/s00213-020-05543-7 32435819

[B17] Endoh T. Matsuura H. Tanaka C. Nagase H. (1992). Nor-binaltorphimine: a Potent and Selective Kappa-Opioid Receptor Antagonist with Long-Lasting Activity *In Vivo* . Arch. Int. Pharmacodyn Ther. 316, 30–42. 1326932

[B18] Ewertz M. Qvortrup C. Eckhoff L. (2015). Chemotherapy-induced Peripheral Neuropathy in Patients Treated with Taxanes and Platinum Derivatives. Acta Oncol. 54 (5), 587–591. 10.3109/0284186X.2014.995775 25751757

[B19] Ferrari L. F. Araldi D. Green P. G. Levine J. D. (2020). Marked Sexual Dimorphism in Neuroendocrine Mechanisms for the Exacerbation of Paclitaxel-Induced Painful Peripheral Neuropathy by Stress. Pain 161 (4), 865–874. 10.1097/j.pain.0000000000001798 31917777 PMC7085433

[B20] Finnerup N. B. Attal N. Haroutounian S. McNicol E. Baron R. Dworkin R. H. (2015). Pharmacotherapy for Neuropathic Pain in Adults: a Systematic Review and Meta-Analysis. Lancet Neurol. 14 (2), 162–173. 10.1016/S1474-4422(14)70251-0 25575710 PMC4493167

[B21] Flatters S. J. Bennett G. J. (2004). Ethosuximide Reverses Paclitaxel- and Vincristine-Induced Painful Peripheral Neuropathy. Pain 109 (1-2), 150–161. 10.1016/j.pain.2004.01.029 15082137

[B22] Forman A. (1990). Peripheral Neuropathy in Cancer Patients: Clinical Types, Etiology, and Presentation. Part 2. Oncology (Williston Park) 4 (2), 85–89. 2167114

[B23] Freye E. Hartung E. Schenk G. K. (1983). Bremazocine: an Opiate that Induces Sedation and Analgesia without Respiratory Depression. Anesth. Analg 62 (5), 483–488. 10.1213/00000539-198305000-00005 6301311

[B24] Grace P. M. Strand K. A. Galer E. L. Urban D. J. Wang X. Baratta M. V. (2016). Morphine Paradoxically Prolongs Neuropathic Pain in Rats by Amplifying Spinal NLRP3 Inflammasome Activation. Proc. Natl. Acad. Sci. U S A. 113 (24), E3441–E3450. 10.1073/pnas.1602070113 27247388 PMC4914184

[B25] Hershman D. L. Lacchetti C. Dworkin R. H. Lavoie Smith E. M. Bleeker J. Cavaletti G. (2014). Prevention and Management of Chemotherapy-Induced Peripheral Neuropathy in Survivors of Adult Cancers: American Society of Clinical Oncology Clinical Practice Guideline. J. Clin. Oncol. 32 (18), 1941–1967. 10.1200/JCO.2013.54.0914 24733808

[B26] Holmes F. A. Walters R. S. Theriault R. L. Forman A. D. Newton L. K. Raber M. N. (1991). Phase II Trial of Taxol, an Active Drug in the Treatment of Metastatic Breast Cancer. J. Natl. Cancer Inst. 83 (24), 1797–1805. 10.1093/jnci/83.24.1797-a 1683908

[B27] Hwang B. Y. Kim E. S. Kim C. H. Kwon J. Y. Kim H. K. (2012). Gender Differences in Paclitaxel-Induced Neuropathic Pain Behavior and Analgesic Response in Rats. Korean J. Anesthesiol 62 (1), 66–72. 10.4097/kjae.2012.62.1.66 22323957 PMC3272532

[B28] Jaggi A. S. Jain V. Singh N. (2011). Animal Models of Neuropathic Pain. Fundam. Clin. Pharmacol. 25 (1), 1–28. 10.1111/j.1472-8206.2009.00801.x 20030738

[B29] Khanna C. Rosenberg M. Vail D. M. (2015). A Review of Paclitaxel and Novel Formulations Including Those Suitable for Use in Dogs. J. Vet. Intern. Med. 29 (4), 1006–1012. 10.1111/jvim.12596 26179168 PMC4895360

[B30] Kishioka S. Kiguchi N. Kobayashi Y. Yamamoto C. Saika F. Wakida N. (2013). Pharmacokinetic Evidence for the Long-Lasting Effect of Nor-Binaltorphimine, a Potent Kappa Opioid Receptor Antagonist, in Mice. Neurosci. Lett. 552, 98–102. 10.1016/j.neulet.2013.07.040 23933210

[B31] Lee S. K. (2018). Sex as an Important Biological Variable in Biomedical Research. BMB Rep. 51 (4), 167–173. 10.5483/bmbrep.2018.51.4.034 29429452 PMC5933211

[B32] Loprinzi C. L. Lacchetti C. Bleeker J. Cavaletti G. Chauhan C. Hertz D. L. (2020). Prevention and Management of Chemotherapy-Induced Peripheral Neuropathy in Survivors of Adult Cancers: ASCO Guideline Update. J. Clin. Oncol. 38 (28), 3325–3348. 10.1200/JCO.20.01399 32663120

[B33] McLaughlin J. P. Myers L. C. Zarek P. E. Caron M. G. Lefkowitz R. J. Czyzyk T. A. (2004). Prolonged Kappa Opioid Receptor Phosphorylation Mediated by G-Protein Receptor Kinase Underlies Sustained Analgesic Tolerance. J. Biol. Chem. 279 (3), 1810–1818. 10.1074/jbc.M305796200 14597630 PMC2131729

[B34] Meade J. A. Alkhlaif Y. Contreras K. M. Obeng S. Toma W. Sim-Selley L. J. (2020). Kappa Opioid Receptors Mediate an Initial Aversive Component of Paclitaxel-Induced Neuropathy. Psychopharmacology (Berl) 237 (9), 2777–2793. 10.1007/s00213-020-05572-2 32529265

[B35] Mogil J. S. (2012). Sex Differences in Pain and Pain Inhibition: Multiple Explanations of a Controversial Phenomenon. Nat. Rev. Neurosci. 13 (12), 859–866. 10.1038/nrn3360 23165262

[B36] Mols F. Beijers T. Vreugdenhil G. van de Poll-Franse L. (2014). Chemotherapy-induced Peripheral Neuropathy and its Association with Quality of Life: a Systematic Review. Support Care Cancer 22 (8), 2261–2269. 10.1007/s00520-014-2255-7 24789421

[B37] Munro T. A. Rizzacasa M. A. (2003). Salvinorins D-F, New Neoclerodane Diterpenoids from Salvia Divinorum, and an Improved Method for the Isolation of Salvinorin A. J. Nat. Prod. 66 (5), 703–705. 10.1021/np0205699 12762813

[B38] Naji-Esfahani H. Vaseghi G. Safaeian L. Pilehvarian A. A. Abed A. Rafieian-Kopaei M. (2016). Gender Differences in a Mouse Model of Chemotherapy-Induced Neuropathic Pain. Lab. Anim. 50 (1), 15–20. 10.1177/0023677215575863 25732574

[B39] Obara I. Mika J. Schafer M. K. Przewlocka B. (2003). Antagonists of the Kappa-Opioid Receptor Enhance Allodynia in Rats and Mice after Sciatic Nerve Ligation. Br. J. Pharmacol. 140 (3), 538–546. 10.1038/sj.bjp.0705427 12970097 PMC1574046

[B40] Paton K. F. Atigari D. V. Kaska S. Prisinzano T. Kivell B. M. (2020a). Strategies for Developing κ Opioid Receptor Agonists for the Treatment of Pain with Fewer Side Effects. J. Pharmacol. Exp. Ther. 375 (2), 332–348. 10.1124/jpet.120.000134 32913006 PMC7589957

[B41] Paton K. F. Biggerstaff A. Kaska S. Crowley R. S. La Flamme A. C. Prisinzano T. E. (2020b). Evaluation of Biased and Balanced Salvinorin A Analogs in Preclinical Models of Pain. Front. Neurosci. 14 (765), 765. 10.3389/fnins.2020.00765 32792903 PMC7385413

[B42] Paton K. F. Kumar N. Crowley R. S. Harper J. L. Prisinzano T. E. Kivell B. M. (2017). The Analgesic and Anti-inflammatory Effects of Salvinorin A Analogue β-tetrahydropyran Salvinorin B in Mice. Eur. J. Pain 21 (6), 1039–1050. 10.1002/ejp.1002 28158929 PMC5466480

[B43] Pattinson K. T. (2008). Opioids and the Control of Respiration. Br. J. Anaesth. 100 (6), 747–758. 10.1093/bja/aen094 18456641

[B44] Pieretti S. Di Giannuario A. Di Giovannandrea R. Marzoli F. Piccaro G. Minosi P. (2016). Gender Differences in Pain and its Relief. Ann. Ist Super Sanita 52 (2), 184–189. 10.4415/ANN_16_02_09 27364392

[B45] Porreca F. Mosberg H. I. Hurst R. Hruby V. J. Burks T. F. (1984). Roles of Mu, delta and Kappa Opioid Receptors in Spinal and Supraspinal Mediation of Gastrointestinal Transit Effects and Hot-Plate Analgesia in the Mouse. J. Pharmacol. Exp. Ther. 230 (2), 341–348. 6086883

[B46] Prevatt-Smith K. M. Lovell K. M. Simpson D. S. Day V. W. Douglas J. T. Bosch P. (2011). Potential Drug Abuse Therapeutics Derived from the Hallucinogenic Natural Product Salvinorin A. Medchemcomm 2 (12), 1217–1222. 10.1039/C1MD00192B 22442751 PMC3307802

[B47] Rahn E. J. Zvonok A. M. Thakur G. A. Khanolkar A. D. Makriyannis A. Hohmann A. G. (2008). Selective Activation of Cannabinoid CB2 Receptors Suppresses Neuropathic Nociception Induced by Treatment with the Chemotherapeutic Agent Paclitaxel in Rats. J. Pharmacol. Exp. Ther. 327 (2), 584–591. 10.1124/jpet.108.141994 18664590 PMC2682949

[B48] Rasakham K. Liu-Chen L. Y. (2011). Sex Differences in Kappa Opioid Pharmacology. Life Sci. 88 (1-2), 2–16. 10.1016/j.lfs.2010.10.007 20951148 PMC3870184

[B49] Riley A. P. Groer C. E. Young D. Ewald A. W. Kivell B. M. Prisinzano T. E. (2014). Synthesis and κ-opioid Receptor Activity of Furan-Substituted Salvinorin A Analogues. J. Med. Chem. 57 (24), 10464–10475. 10.1021/jm501521d 25426797 PMC4281103

[B50] Roeckel L. A. Le Coz G. M. Gavériaux-Ruff C. Simonin F. (2016). Opioid-induced Hyperalgesia: Cellular and Molecular Mechanisms. Neuroscience 338, 160–182. 10.1016/j.neuroscience.2016.06.029 27346146

[B51] Rowinsky E. K. Chaudhry V. Forastiere A. A. Sartorius S. E. Ettinger D. S. Grochow L. B. (1993). Phase I and Pharmacologic Study of Paclitaxel and Cisplatin with Granulocyte colony-stimulating Factor: Neuromuscular Toxicity Is Dose-Limiting. J. Clin. Oncol. 11 (10), 2010–2020. 10.1200/JCO.1993.11.10.2010 7692001

[B52] Schechter M. D. (1997). Discrete versus Cumulative Dosing in Dose-Response Discrimination Studies. Eur. J. Pharmacol. 326 (2-3), 113–118. 10.1016/s0014-2999(97)85404-0 9196262

[B53] Schmidt M. D. Schmidt M. S. Butelman E. R. Harding W. W. Tidgewell K. Murry D. J. (2005). Pharmacokinetics of the Plant-Derived Kappa-Opioid Hallucinogen Salvinorin A in Nonhuman Primates. Synapse 58 (3), 208–210. 10.1002/syn.20191 16138318

[B54] Seretny M. Currie G. L. Sena E. S. Ramnarine S. Grant R. MacLeod M. R. (2014). Incidence, Prevalence, and Predictors of Chemotherapy-Induced Peripheral Neuropathy: A Systematic Review and Meta-Analysis. Pain 155 (12), 2461–2470. 10.1016/j.pain.2014.09.020 25261162

[B55] Shah A. Hoffman E. M. Mauermann M. L. Loprinzi C. L. Windebank A. J. Klein C. J. (2018). Incidence and Disease burden of Chemotherapy-Induced Peripheral Neuropathy in a Population-Based Cohort. J. Neurol. Neurosurg. Psychiatry 89 (6), 636–641. 10.1136/jnnp-2017-317215 29439162 PMC5970026

[B56] Shansky R. M. Murphy A. Z. (2021). Considering Sex as a Biological Variable Will Require a Global Shift in Science Culture. Nat. Neurosci. 24 (4), 457–464. 10.1038/s41593-021-00806-8 33649507 PMC12900283

[B57] Simon-Arceo K. González-Trujano M. E. Coffeen U. Fernández-Mas R. Mercado F. Almanza A. (2017). Neuropathic and Inflammatory Antinociceptive Effects and Electrocortical Changes Produced by Salvia Divinorum in Rats. J. Ethnopharmacol 206, 115–124. 10.1016/j.jep.2017.05.016 28502907

[B58] Sisignano M. Baron R. Scholich K. Geisslinger G. (2014). Mechanism-based Treatment for Chemotherapy-Induced Peripheral Neuropathic Pain. Nat. Rev. Neurol. 10 (12), 694–707. 10.1038/nrneurol.2014.211 25366108

[B59] Slivicki R. A. Iyer V. Mali S. S. Garai S. Thakur G. A. Crystal J. D. (2020). Positive Allosteric Modulation of CB1 Cannabinoid Receptor Signaling Enhances Morphine Antinociception and Attenuates Morphine Tolerance without Enhancing Morphine- Induced Dependence or Reward. Front. Mol. Neurosci. 13 (54), 54. 10.3389/fnmol.2020.00054 32410959 PMC7199816

[B60] Slivicki R. A. Saberi S. A. Iyer V. Vemuri V. K. Makriyannis A. Hohmann A. G. (2018). Brain-Permeant and -Impermeant Inhibitors of Fatty Acid Amide Hydrolase Synergize with the Opioid Analgesic Morphine to Suppress Chemotherapy-Induced Neuropathic Nociception without Enhancing Effects of Morphine on Gastrointestinal Transit. J. Pharmacol. Exp. Ther. 367 (3), 551–563. 10.1124/jpet.118.252288 30275151 PMC6246979

[B61] Smith S. B. Crager S. E. Mogil J. S. (2004). Paclitaxel-induced Neuropathic Hypersensitivity in Mice: Responses in 10 Inbred Mouse Strains. Life Sci. 74 (21), 2593–2604. 10.1016/j.lfs.2004.01.002 15041441

[B62] Tidgewell K. Harding W. W. Schmidt M. Holden K. G. Murry D. J. Prisinzano T. E. (2004). A Facile Method for the Preparation of Deuterium Labeled Salvinorin A: Synthesis of [2,2,2-2H3]-Salvinorin A. Bioorg. Med. Chem. Lett. 14 (20), 5099–5102. 10.1016/j.bmcl.2004.07.081 15380207

[B63] Tiwari V. Anderson M. Yang F. Tiwari V. Zheng Q. He S. Q. (2018). Peripherally Acting μ-Opioid Receptor Agonists Attenuate Ongoing Pain-Associated Behavior and Spontaneous Neuronal Activity after Nerve Injury in Rats. Anesthesiology 128 (6), 1220–1236. 10.1097/ALN.0000000000002191 29601322 PMC5953805

[B64] Uniyal A. Gadepalli A. Kotiyal A. Tiwari V. (2020). Underpinning the Neurobiological Intricacies Associated with Opioid Tolerance. ACS Chem. Neurosci. 11 (6), 830–839. 10.1021/acschemneuro.0c00019 32083459

[B65] van den Bent M. J. van Raaij-van den Aarssen V. J. Verweij J. Doorn P. A. Sillevis Smitt P. A. (1997). Progression of Paclitaxel-Induced Neuropathy Following Discontinuation of Treatment. Muscle Nerve 20 (6), 750–752. 10.1002/(sici)1097-4598(199706)20:6<750:aid-mus15>3.0.co;2-y 9149085

[B66] Vonvoigtlander P. F. Lahti R. A. Ludens J. H. (1983). U-50,488: a Selective and Structurally Novel Non-mu (Kappa) Opioid Agonist. J. Pharmacol. Exp. Ther. 224 (1), 7–12. 6129321

[B67] Wang Y. C. Li N. Zhao Y. Zhang L. J. (2018). Effects of Female Sex Hormones on Chemotherapeutic Paclitaxel-Induced Neuropathic Pain and Involvement of Inflammatory Signal. J. Biol. Regul. Homeost Agents 32 (5), 1157–1163. 30334407

[B68] Xu M. Petraschka J. P. McLaughlin M. G. Czyzyk T. A. Termanr G. W. Chavkin C. (2004). Neuropathic Pain Activates the Endogenous Kappa Opioid System in Mouse Spinal Cord and Induces Opioid Receptor Tolerance. J. Neurosci. 24 (19), 4576–4584. 10.1523/JNEUROSCI.5552-03.2004 15140929 PMC2376823

